# Editorial: How can We Co-Create Solutions in Health Promotion With Users and Stakeholders?

**DOI:** 10.3389/fpubh.2021.773907

**Published:** 2021-12-08

**Authors:** Christiane Stock, Sonia Dias, Timo Dietrich, Annika Frahsa, Ines Keygnaert

**Affiliations:** ^1^Charité—Universitätsmedizin Berlin, Corporate Member of Freie Universität Berlin and Humboldt-Universität zu Berlin, Institute of Health and Nursing Science, Berlin, Germany; ^2^Unit for Health Promotion Research, University of Southern Denmark, Esbjerg, Denmark; ^3^NOVA National School of Public Health, Public Health Research Centre, Universidade NOVA de Lisboa and Comprehensive Health Research Center (CHRC), Lisbon, Portugal; ^4^Social Marketing @ Griffith, Griffith Business School, Griffith University, Southport, QLD, Australia; ^5^Institute of Social and Preventive Medicine, University of Bern, Bern, Switzerland; ^6^Faculty of Public Health and Primary Care, International Centre for Reproductive Health, Ghent University, Ghent, Belgium

**Keywords:** co-creation, health promotion, participatory research, co-design, stakeholder engagement

## Introduction

Participatory approaches have become an integral part in various fields of public health and health promotion research. These approaches have the potential to allow the production of deeper knowledge of complex health issues by valuing and incorporating the different perspectives and experiences of key actors closely related to the subject of the research (1). The hallmark of participatory research is the establishment of equitable research partnerships with a diverse group of stakeholders such as public health professionals, health activists, government officials, and citizens (2, 3). Participatory or co-creation approaches serve as a guiding principle to ensure stakeholder engagement throughout all the stages of the research and program development phases including developing, refining, and implementing. Originally, co-creation is a concept from management science and software design and is focused on achieving synergistic effects through user participation in the design processes. Co-creation in health promotion aims to improve the life of those who are subjects of research by empowering them to contribute to the research process and outcomes to better advocate for transformative initiatives and changes in public policies that address their health needs (4–7). Such participation asks for a systematic reflection of underlying power relations in the research process through dialog, recursive methods of understanding, joint planning, and co-design. However, reaching a high level of participation from a variety of stakeholders in health research is an exigent process that requires monetary and non-monetary resources. Although both the academic researchers and community co-researchers are considered capable of contributing to knowledge building, often ensuring that all the parties are fully involved in the research process is a hurdle (8) and stakeholder engagement is required (9).

## Co-Creation in Health Promotion

Co-creation is an umbrella term similar to that of participatory design (10). Co-creation is linked to a wide array of methods, among those co-design and co-production (11), but also other approaches such as design thinking, cooperative planning (3, 12), and living lab formats (Dietrich et al.). In health and community settings, co-creation is often depicted as a model of participatory research (13), while others regard co-creation as comprising both the community-based participatory research and integrated knowledge translation (14). In other words, co-creation refers to any act of collective creativity with a wide range of methods and processes on how this can be achieved. However, to this day only, few methodologies detail how to incorporate stakeholders and citizens values with scientific evidence. In order to overcome the challenges for developers of innovative programs, interventions, or services in the field of health promotion and education, more research is needed to articulate and document essential factors contributing to successful co-creation processes.

A useful specification of the co-creation of knowledge definition was recently delivered by Pearce et al. (11) based on a content analysis of existing studies that involve co-creation. The authors have distinguished four collaborative stages of co-creation research, namely, generating an idea (co-ideation); designing the program or policy and the research methods (co-design); implementing the program or policy according to the agreed upon research methods (co-implementation); and the collection, analysis, and interpretation of data (co-evaluation). This special issue presents innovative research in all the four stages of co-creation. We hope you enjoy reading the articles that are briefly described as follows.

## Articles in the Collection

Addressing the co-ideation stage, the article by Dias et al. outlines the protocol for a migrant community-based project that seeks to optimize health literacy, health promotion, and social cohesion in support of prevention of non-communicable diseases (NCDs) among migrants. This protocol is an example of co-ideation, which is guided by a grounded approach to produce evidence on health literacy needs from key stakeholders and migrant communities. As another article positioned at the co-ideation stage, Choi et al. conducted a mixed method study with teachers and students to understand the needs of school health priorities for rural areas in Peru. This study serves as a starting point to develop a school health program and identifies important priorities that must be considered when designing for remote areas. Also, Onasanya et al. conducted a qualitative study based on key informant interviews and focus group discussions to identify relevant stakeholders for schistosomiasis diagnostics in South-West Nigeria. This study presents a systematic approach to identify stakeholders and classify them into a power/interest matrix according prior to starting a co-creation and co-implementation process.

Three articles address the co-creation stage and focus on collaborative involvement in health intervention design. Dietrich et al. draw from two case studies where researchers co-created virtual reality interventions in an alcohol prevention context. They explore and reflect on two co-creation methods—co-design and living lab—and showcase the different procedures of each approach along with a discussion on the challenges and merits. Ferschl et al. report result from a transdisciplinary research consortium on scientific cooperation and the co-production of scientific outcomes for physical activity promotion. Cheng et al. apply a systematic approach of community co-design to the digital context to generate solutions to improve health and equity outcomes.

Addressing co-implementation, Minian et al. analyze a co-creation process between researchers and patients with lived experiences to co-design resources that encourage behavior change among treatment-seeking smokers. This study can serve as an example of how integrating patients into the planning and delivery of healthcare can contribute to more tailored and effective communication resources. Another article by Kwon et al. analyzes the lessons learned from a case study of school health in a community-based school reopening during coronavirus disease 2019 (COVID-19) pandemic.

Three articles address specific outcomes of co-creation projects. Anang et al. illustrate the lessons learned from “Building on Strengths in Naujaat,” a resiliency initiative with the objective of promoting sense of belonging, collective efficacy, and well-being in Inuit youth. While their creativity and resourcefulness are at the heart of the initiative, this study explores conflicts and pitfalls that accompanied it. von Heimburg et al. explore, using kindergartens as a case setting, how participatory action research can be a tool for transformative practices in a local community. This study shows that how cycles of transformative actions and reflections in co-creation processes bear potential for social inclusion and ultimately for achieving well-being among different stakeholder groups in early childhood development. The other article by Ruiz-Eugenio et al. is a qualitative study on dialogic literary gatherings as co-creation intervention and evaluates its impact on psychological and social well-being in women during COVID-19 lockdown.

## Author Contributions

CS formulated a draft. SD, TD, AF, and IK revised the manuscript. All authors contributed to the article and approved the submitted version.

## Conflict of Interest

The authors declare that the research was conducted in the absence of any commercial or financial relationships that could be construed as a potential conflict of interest.

## Publisher's Note

All claims expressed in this article are solely those of the authors and do not necessarily represent those of their affiliated organizations, or those of the publisher, the editors and the reviewers. Any product that may be evaluated in this article, or claim that may be made by its manufacturer, is not guaranteed or endorsed by the publisher.
